# Prognostic and diagnostic value of circRNA expression in cervical cancer: a meta analysis

**DOI:** 10.3389/fonc.2024.1488040

**Published:** 2025-01-13

**Authors:** Yi Xu, Changxia Li, Lifang Cheng, Shaoheng Wang, Yuqing Wu, Shiyang Li, Mingqiang Liu, Xiaohua Tao

**Affiliations:** ^1^ Dermatology Hospital of Jiangxi Province, Jiangxi Provincial Clinical Research Center For Skin Diseases, Candidate Branch of National Clinical Research Center for Skin Diseases, Dermatology Institute of Jiangxi Province, The Affiliated Dermatology Hospital of Nanchang University, Nanchang, China; ^2^ Third Clinical Medical College, Nanchang University, Nanchang, China

**Keywords:** cervical cancer, circular RNAs, meta-analysis, diagnosis, prognosis, clinicopathological

## Abstract

**Introduction:**

Cervical cancer (CC) is a highly prevalent malignancy of the reproductive system. This study aimed to methodically assess the function of circular RNAs (circRNAs) as possible indicators of CC, with a specific emphasis on their usefulness in the identification, prediction, and correlation with clinicopathological elements.

**Methods:**

A comprehensive literature search was conducted using databases such as PubMed, Cochrane Library, Web of Science, Embase, and the China National Knowledge Infrastructure (CNKI). The latest data were extracted on May 3^rd^, 2024. The diagnostic potential of circRNA expression was evaluated using a range of metrics including sensitivity, specificity, and area under the receiver operating characteristic curve (AUC). The importance of circRNAs was further evaluated in terms of clinical relevance, pathological features, and prognostic value using pooled odds ratios (ORs) and hazard ratios (HRs).

**Results:**

The meta-analysis included 27 studies, which were categorised based on diagnostic applications (n=3), clinicopathological correlations (n=15), and prognostic evaluations (n=23). Elevated expression levels of oncogenic circRNAs were significantly associated with poor clinical indicators, including tumour size (odds ratio [OR] = 0.425, 95% confidence interval [CI]: 0.267–0.676), International Federation of Gynaecology and Obstetrics (FIGO) stage (OR = 0.315, 95% CI: 0.224–0.443), and lymph node metastasis (OR = 2.975, 95% CI: 1.816–4.872). This upregulation of oncogenic circRNA was also identified as a predictor of worse survival outcomes, with a hazard ratio (HR) of 2.13 (95% CI: 1.73–2.62, *P* < 0.001). The downregulation of circRNAs with tumour-suppressor properties was similarly associated with poor clinical parameters, such as tumour size (OR = 0.310, 95% CI: 0.102–0.941), FIGO stage (OR = 0.231, 95% CI: 0.101–0.527), and lymph node metastasis (OR = 2.430, 95% CI: 1.156–5.110), and was indicative of a worsened prognosis (HR = 2.20, 95% CI: 1.03–4.70, *P* = 0.042). In terms of diagnostic value, the pooled sensitivity, specificity, and area under the curve (AUC) were calculated to be 0.85, 0.83, and 0.91, respectively.

**Conclusion:**

The results of our meta-analysis indicate that circRNAs have the potential to serve as promising biomarkers for CC diagnosis, prognosis, and clinicopathology.

**Systematic review registration:**

https://www.crd.york.ac.uk/PROSPERO/, identifier CRD42024544997.

## 1 Introduction

Cervical cancer (CC), predominantly caused by high-risk human papillomavirus (HPV) infection, is the fourth most common malignancy in women worldwide (1). It was associated with approximately 660,000 new cases and 350,000 deaths in 2022 alone, disproportionately affecting women in low- and middle-income areas and those who were human immunodeficiency virus (HIV)-positive (2). In recent years, China has experienced a troubling increase in the incidence of CC, characterised by increasing prevalence and a shift toward younger age groups. This trend contrasts with global patterns, which are influenced by disparate levels of healthcare access and screening programs (3, 4). In the international medical community, the standard approach for the diagnosis and treatment of CC is well-established and includes a combination of screening, surgical intervention, radiotherapy, and chemotherapy. Screening typically involves Pap smear and HPV DNA testing, which significantly reduces the incidence of CC in areas with robust screening programs (5). When CC is detected, the FIGO (International Federation of Gynecology and Obstetrics) staging system is commonly used to guide treatment decisions (6). Early stage CC is often treated surgically, including radical hysterectomy and pelvic lymph node dissection (7). Advanced cancer stages may require concurrent chemoradiation, in which radiation therapy is combined with chemotherapy to enhance the radiosensitivity of cancer cells (8). The 5-year survival rate for CC varies according to the stage at diagnosis, with higher rates for early stage disease (approximately 92% for stage I) and lower rates for later stages (approximately 56% for stage IV) (9). These findings highlight the importance of early detection and treatment. Therefore, it is imperative to develop biomarkers that can facilitate early diagnosis and enable the accurate assessment of prognosis in individuals with CC.

Circular RNAs (circRNAs) are non-coding RNA that create covalently closed continuous loops. These loops control gene expression by silencing miRNAs or attaching proteins that influence cellular processes (10). Recently, circRNAs have garnered considerable attention owing to their role in tumour biology as crucial regulators of gene expression and cellular function. For example, specific circRNAs have been shown to modulate the miR-187-3p/RTKN2 pathway in hepatocellular carcinoma, thereby promoting tumour growth and metastasis (11). Similarly, in glioblastomas, circRNAs can act as miRNAs, affecting the expression of genes pivotal to cell proliferation and survival (12). These examples underscore the multifaceted influence of circRNAs in shaping the tumour microenvironment and highlight their potential as biomarkers and therapeutic targets.

CircRNAs have been acknowledged as important regulators of CC. For example, Zhang et al. (13) showed that circCDKN2B-AS1 enhances aerobic glycolysis by absorbing IMP3 protein to stabilise HK2 mRNA, enhancing the malignant characteristics of CC, which may offer a prospective strategy for CC treatment. Abnormal expression of circRNAs such as circSLC26A4 and circ-ATP8A2 has been linked to the promotion of CC progression through the miR-1287-5p/HOXA7 axis and other molecular mechanisms (14). These findings highlight the potential of circRNAs as biomarkers for early diagnosis and as therapeutic targets for intervention in CC. However, these studies had several limitations. First, many of the available studies had small sample sizes, limiting the generalisability and reliability of the findings. Second, most studies focused on specific types of circRNAs, whereas the roles of other types of circRNAs in CC have not yet been fully explored. This limits our understanding of the role of circRNAs in CC.

In this meta-analysis, we conducted a comprehensive review of the literature on the role of circRNAs in CC, with particular focus on their potential as diagnostic, clinicopathological, and prognostic biomarkers. The primary objective of this study was to quantitatively synthesise available data to assess the effectiveness of circRNAs in the above contexts. We sought to reconcile the heterogeneity among studies, evaluate the methodological rigor of the included studies, and scrutinise potential publication biases that could affect the robustness of our conclusions. Here, we provide a comprehensive and systematic assessment of the role of circRNAs in CC, which will contribute to the refinement of personalised treatment strategies and enhance the clinical management of affected patients.

## 2 Materials and methods

### 2.1 Registration

The methodology of this study was meticulously pre-registered on the PROSPERO platform under registration number CRD42024544997. This ensured transparency and reproducibility of our research.

### 2.2 Data search strategy

The literature review was conducted in compliance with the PRISMA (Preferred Reporting Items for Systematic Reviews and Meta-Analyses) statement.

The databases PubMed, Cochrane Library, Web of Science, Embase, and China National Knowledge Infrastructure (CNKI) were searched for scholarly articles on circRNAs and CC, focusing on publications up to the date of May 3^rd^, 2024.

To ensure comprehensiveness, literature retrieval was performed by two researchers working independently using both Medical Subject Headings (MeSH) and free-text keywords. In case of divergence, a third researcher’s input was sought. The search strategy encompassed the following MeSH terms and keywords (1): “Uterine Cervical Neoplasms”, “Cervical Cancer”, and “Cancer of the Cervix”; (2) “Circular RNA”, “CiRNA”, and “circRNAs”. The authors of relevant articles were contacted for further information when necessary.

### 2.3 Inclusion and exclusion criteria

Two researchers, working independently, evaluated the suitability of the studies and gathered relevant data. Any conflicts were addressed through consultation with an additional researcher. Studies that explored the significance of circRNA expression levels in relation to CC prognosis, diagnosis, and clinicopathological features were deemed eligible for meta-analysis.

The inclusion criteria were as follows: (1) studies in which included participants were diagnosed as having CC using pathology, (2) studies presenting circRNA levels categorised as either elevated or diminished, (3) research encompassing data to assess diagnostic, prognostic, and clinicopathological aspects, and (4) investigations of a cohort or case-control design.

The exclusion criteria were as follows: (1) redundant studies that constituted duplicates; (2) publications falling under the categories of reviews, meta-analyses, correspondence pieces, abstracts from conferences, or individual case reports; and (3) research endeavours with ambiguous or insufficient data, which precludes the conduct of an appropriate statistical analysis.

### 2.4 Data extraction

Two researchers independently gathered data from the selected studies, adhering to consistent criteria. The following data were collected: (1) prognostic data, including the expression level, threshold value, sample size, assay method, duration of follow-up, survival results, and survival results, including the hazard ratios (HRs) of overall survival (OS) or disease-free survival (DFS) and 95% confidence intervals (CIs). (2) Diagnostic data, including odds ratios (ORs) and 95% CIs, were used to integrate information and assess diagnostic accuracy. This was achieved using the following parameters: true positives (TP), false positives (FP), false negatives (FN), true negatives (TN), and the area under the receiver operating characteristic curve (ROC) (AUC) of each study. This enabled the evaluation of sensitivity (SEN) and specificity (SPE). (3) The clinicopathological characteristics included age, tumour size, FIGO stage, lymph node metastasis, and tumour differentiation. In instances where the HRs and their 95% CIs were not directly reported, these data were extracted from Kaplan–Meier (KM) curves using the Engauge Digitiser software, version 4.1. HRs and the accompanying 95% CIs were subsequently derived using the Excel program provided by Tierney et al. (15). In instances where TP, TN, FP, and FN were not explicitly stated, these metrics were inferred from the SEN and SPE values of the ROC curve facilitated by the GetData Graph Digitizer software (16).

### 2.5 Quality assessment

Quality assessment was independently conducted by two researchers, with the input of a third researcher in cases of discrepancy. The methodological quality of the included diagnostic studies was evaluated in accordance with the four domains of the Diagnostic Accuracy Studies-2(QUADAS-2)tool: patient selection, index tests employed, reference standards applied, and flow and timing of the study design. The risk of bias in these domains was classified into three categories: high (H), low (L), or unclear (U). The quality of the prognostic studies was evaluated using the Newcastle-Ottawa Scale (NOS). A score of 7 or higher was considered indicative of a high level of methodological quality.

### 2.6 Statistical analysis

Statistical analysis was conducted using Review Manager 5.3 and Stata 12.0 software. The degree of heterogeneity among the included studies was evaluated using the *I²* statistic. A threshold of *I²* greater than 50%, in conjunction with a *P*-value of < 0.05, was established to indicate significant heterogeneity. In such instances, a random-effects model was used to aggregate the study results. Conversely, in instances where heterogeneity was deemed insignificant, a fixed-effects model was employed for the analysis. The threshold for statistical significance was set at a *P*-value of less < 0.05.

In the diagnostic meta-analysis, the TP, FP, FN, and TN were combined to calculate the pooled SEN and SPE of circRNA expression. The diagnostic accuracy was evaluated by calculating the area under the summary ROC (SROC). Deeks’ funnel plot asymmetry test was used to examine the potential for publication bias. A P value exceeding 0.1 indicates the absence of publication bias. In the prognostic meta-analysis, HRs and their corresponding 95% confidence intervals (CIs) were used to quantify the prognostic significance of the circRNAs. The presence of publication bias was evaluated using Egger’s test, with a P-value greater than 0.1 indicating the absence of such bias. Sensitivity analysis was conducted to ensure the robustness of the pooled HRs. Finally, pooled ORs and 95% CIs were calculated to investigate the correlation between circRNA expression levels and various clinicopathological features.

## 3 Results

### 3.1 Search results

**Figure 1:**
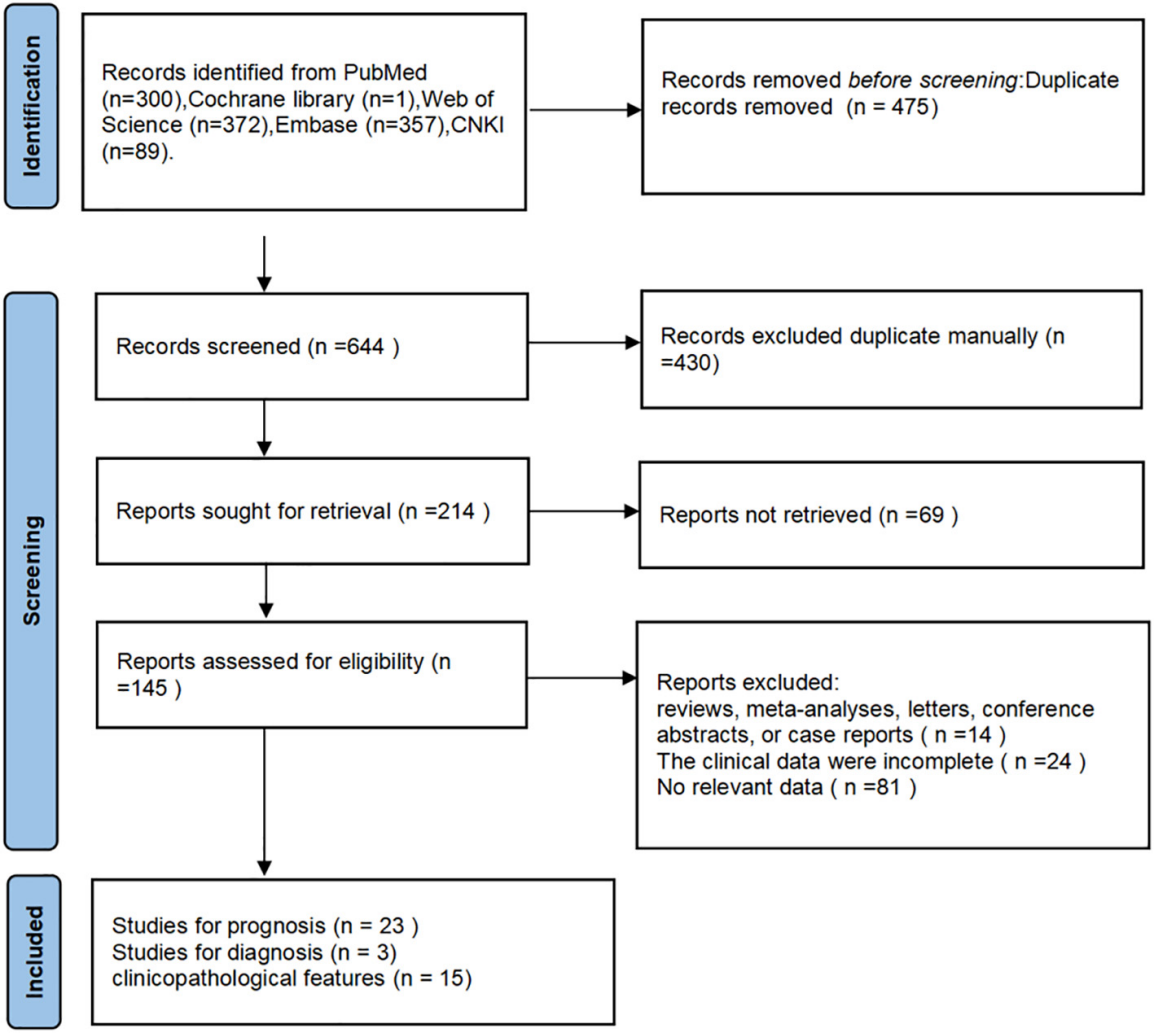
The PRISMA flowchart of the literature selection process.

The literature search and screening processes used in this study are illustrated in **Figure 1**. The preliminary search yielded 1,119 potentially relevant studies from various databases, including PubMed (300 articles), Cochrane Library (one article), Web of Science (372 articles), Embase (357 articles), and CNKI (89 articles). In total, 214 unique articles were identified after removing duplicate records. Of the aforementioned studies, the full texts of 69 were not accessible. After comprehensively reviewing the remaining full-text articles, 113 were excluded. Fourteen studies were not case-control or cohort studies, 24 lacked comprehensive clinical data, and 81 did not provide the data required for analysis. A total of 27 studies published between 2018 and 2023 were selected for inclusion in the meta-analysis, which was conducted using the following categories: prognostic analyses (17–39), diagnostic assessments (21, 40, 41), and clinicopathological features (17, 18, 20, 22, 24, 28–36, 42).

### 3.2 Study characteristics and quality assessment

**Tables 1**–**4** present the main characteristics of the mentioned studies. Between 2018 and 2023, 27 circRNAs were identified and published. The studies that had been included were all from China. A total of 23 circRNAs were used in 23 investigations (**Table 1**), offering fundamental data for prognostic analysis. The duration of the patients’ follow-up varied from 36 to 140 months, and 20 to 192 samples were taken. On each of the nine dimensions, the NOS scores demonstrated the strength of the included studies for prognostic and clinical parameter analysis (**Table 2**).

**Table 1:** Major distinguishing features of the prognostic studies included in the meta-analysis.

No	Study	Year	Country	Sample size	Circ	CircRNA expression		Detection method	Regulation	Follow up time (month)	Survival analysis	Survival outcome	Variables			
						High	Low						HR	95%CI	P value	Obtained
1	Jing He (21)	2020	China	192	circ_0018289	96	96	qRT-PCR	upregulated	48	Univa	DFS	2.198	1.254-3.853	0.006	Direct
2	Jing He (21)	2020	China	192	circ_0018289	96	96	qRT-PCR	upregulated	48	Multiva	DFS	1.816	1.022-3.226	0.042	Direct
3	Jing He (21)	2020	China	192	circ_0018289	96	96	qRT-PCR	upregulated	48	Univa	OS	2.265	1.151-4.457	0.018	Direct
4	Liquan Chen (17)	2020	China	50	circ_0084927	25	25	qRT-PCR	upregulated	60	Univa	OS	1.64	0.52-5.16	0.0054	KM
5	Lin Ding (18)	2019	China	46	circ-ATP8A2	25	21	qRT-PCR	upregulated	60	Univa	OS	1.69	0.54-5.31	0.007	KM
6	Yifan Mao (28)	2019	China	20	circEIF4G2	10	10	qRT-PCR	upregulated	60	Univa	OS	1.68	0.07-40.27	<0.05	KM
7	Zhizhen Wang (33)	2021	China	68	circMYC	34	34	qRT-PCR	upregulated	60	Univa	OS	2.47	0.58-10.49	<0.05	KM
8	Heli Li (25)	2023	China	84	circPRMT5	44	40	qRT-PCR	upregulated	36	Univa	OS	2. 673	1.581-4.520	<0. 001	Direct
9	Heli Li (25)	2023	China	84	circPRMT5	44	40	qRT-PCR	upregulated	36	Multiva	OS	2. 512	1.533-4.116	<0. 001	Direct
10	Lilan Wu (34)	2023	China	78	circ_0000118	39	39	qRT-PCR	upregulated	48	Univa	OS	0.46	0.02-10.82	0.0385	KM
11	Yan Li (26)	2022	China	40	circ_0001627	21	19	qRT-PCR	upregulated	48	Univa	OS	1.16	0.13-10.23	<0.05	KM
12	Tiefang Song (30)	2018	China	39	circ_101996	20	19	qRT-PCR	upregulated	78	Univa	OS	2.15	0.3-15.5	0.032	KM
13	Suzhen Fan (20)	2020	China	64	circGSE1	34	30	qRT-PCR	upregulated	64	Univa	OS	1.64	0.54-4.99	0.0105	KM
14	Suzhen Fan (20)	2020	China	64	circGSE1	34	30	qRT-PCR	upregulated	64	Univa	DFS	2.75	1.13-6.66	0.0018	KM
15	Fei Ji (23)	2019	China	35	circSLC26A4	19	16	qRT-PCR	upregulated	48	Univa	OS	1.63	0.51-5.19	<0.01	KM
16	Dan-Dan Yuan (36)	2021	China	50	circ_0000730	25	25	qRT-PCR	down-regulated	55	Univa	OS	2.04	0.61-6.84	0.0358	KM
17	Jianhua Zhang (39)	2018	China	53	circ_0023404	27	26	qRT-PCR	upregulated	76	Univa	OS	2.72	0.36-20.68	<0.05	KM
18	Chunyu Zhang1 (38)	2021	China	140	circ_0043280	58	82	qRT-PCR	down-regulated	140	Univa	OS	3.08	0.95-9.95	<0. 001	KM
19	Chunyu Zhang1 (38)	2021	China	140	circ_0043280	58	82	qRT-PCR	down-regulated	140	Univa	DFS	2.65	0.96-7.29	0.0015	KM
20	Yi Ding (19)	2021	China	55	circ_0001772(circRBM33)	28	27	qRT-PCR	upregulated	60	Univa	OS	1.70	0.43-6.74	0.0086	KM
21	Chunyu Zhang (37)	2023	China	94	circ_0065898(circVPRBP)	38	56	qRT-PCR	down-regulated	110	Univa	OS	1.03	0.04-24.02	0.005	KM
22	Chunyu Zhang (37)	2023	China	94	circ_0065898(circVPRBP)	38	56	qRT-PCR	down-regulated	110	Univa	DFS	1.39	0.26-7.42	0.004	KM
23	Peijing Shi (29)	2020	China	46	circ_0084927	23	23	qRT-PCR	upregulated	60	Univa	OS	1.67	0.53-5.22	0.028	KM
24	Zhilan Yao (35)	2021	China	50	circ_000543	25	25	qRT-PCR	upregulated	58	Univa	OS	2.30	0.72-7.36	0.0189	KM
25	Fei Ji (24)	2021	China	48	circARHGAP12	24	24	qRT-PCR	upregulated	60	Univa	OS	1.28	0.17-9.88	<0.05	KM
26	Yiting Wang (32)	2019	China	55	circ_0001038	30	25	qRT-PCR	upregulated	60	Univa	OS	1.52	0.64-3.62	0.03	KM
27	Hanqing Hong (22)	2019	China	48	circCLK3	29	19	qRT-PCR	upregulated	60	Univa	OS	2.59	0.40-16.97	<0.01	KM
28	Hanqing Hong (22)	2019	China	48	circCLK3	29	19	qRT-PCR	upregulated	60	Univa	DFS	1.81	0.16-19.84	<0.01	KM
29	Tian Jun (31)	2021	China	70	circPGAP3	35	35	qRT-PCR	down-regulated	60	Univa	OS	1.30	0.16-10.67	0.015	KM
30	Xuebao Mao (27)	2023	China	180	circ_0010423	95	85	qRT-PCR	upregulated	60	Univa	OS	2.16	1.38-3.38	<0.01	KM

OS, overall survival; DFS, disease-free survival; Multiva, multivariate; Univa, univariate; HR, hazard ratio; 95% CI, 95% confidence interval; KM, KM curve; NA, not available.

**Table 2:** Assessment of qualifying clinical parameter and prognostic research using the Newcastle-Ottawa Scale.

Study	Selection				Comparability		Outcome			Total
	Representativeness of the exposed cohort	Selection of the non exposed cohort	Ascertainment of exposure	Demonstration that outcome of interest was not present at start of study	Controlled the differential expression of circRNAs	Controled any additional factor	Assessment of outcome	Follow-up long enough	Adequacy follow up	
Jing He (21)	1	1	1	1	1	1	1	0	1	8
Liquan Chen (17)	1	1	1	1	1	1	1	1	1	9
Lin Ding (18)	1	0	1	1	1	0	1	1	1	7
YiFan Mao (28)	1	1	1	1	1	1	1	1	1	8
Zhizhen Wang (33)	1	1	1	1	1	0	1	1	1	8
Heli Li (25)	1	1	1	1	1	1	1	0	1	8
Lilan Wu (34)	1	1	1	1	1	0	1	0	1	7
Yan Li (26)	1	1	1	1	1	1	1	0	1	8
Tiefang Song (30)	1	1	1	1	1	1	1	1	1	9
Suzhen Fan (20)	1	1	1	1	1	1	1	1	1	9
Fei Ji (23)	1	1	1	1	1	1	1	0	1	8
Dan-Dan Yuan (36)	1	1	1	1	1	1	1	0	1	8
Jianhua Zhang (39)	1	1	1	1	1	1	1	1	1	9
Chunyu Zhang1 (38)	1	1	1	1	1	1	1	1	1	9
Yi Ding (19)	1	1	1	1	1	1	1	1	1	9
Chunyu Zhang (37)	1	1	1	1	1	0	1	1	1	8
Peijing Shi (29)	1	1	1	1	1	1	1	1	1	9
Zhilan Yao (35)	1	1	1	1	1	1	1	0	1	8
Fei Ji (24)	1	1	1	1	1	1	1	1	1	9
Yiting Wang (32)	1	0	1	1	1	1	1	1	1	8
Hanqing Hong (22)	1	1	1	1	1	1	1	1	1	9
Tian Jun (31)	1	1	1	1	1	1	1	1	1	9
Xuebao Mao (27)	1	1	1	1	1	1	1	1	1	9

**Table 3:** Fundamental characteristics of diagnostic analysis.

Study	Year	Country	CircRNA	Sample size		Methods	Regulation	Diagnosis power		
				Case	Control			Sen	Spe	AUC
Wenyan Liao (40)	2020	China	circ_0107593	52	52	qRT-PCR	downregulated	0.904	0.692	0.869
Mingyi Zhou (41)	2021	China	circZFR	30	30	qRT-PCR	upregulated	0.867	0.867	0.880
Jing He (21)	2020	China	circ_0018289	192	192	qRT-PCR	upregulated	0.807	0.896	0.907

AUC, area under the ROC curve; qRT-PCR, quantitative real-time polymerase chain reaction; Sen, sensitivity; Spe, specificity; CircRNA, circular RNAs.

**Table 4:** Relationship between circRNAs and clinicopathological characteristics in CC.

	Tumor promoter			Tumor Suppressor		
	OR	95%CI	P	OR	95%CI	P
Age (younger/older)	1.093	0.793-1.505	0.588	0.951	0.304-2.975	0.932
Tumor size(≤4/>4cm)	0.425	0.267-0.676	0.000	0.310	0.102-0.941	0.039
FIGO stage(I + II/III + IV)	0.315	0.224 -0.443	0.000	0.231	0.101-0.527	0.000
Lymph node metastasis(Y/N)	2.975	1.816 -4.872	0.000	2.430	1.156-5.110	0.019
Tumor diferentiaion(Well,Moderate/Poor)	0.795	0.422 -1.497	0.477	1.220	0.139-10.726	0.857

CI, confidence interval; OR, odds ratio; N, no; Y, yes.

**Figure 2:**
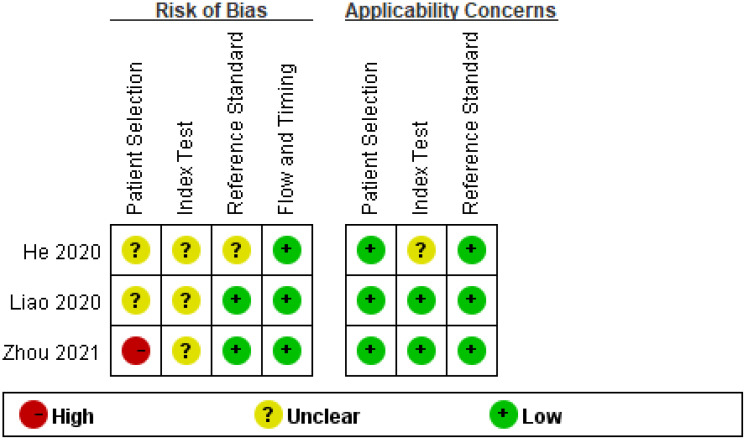
Quality assessment of eligible studies for diagnostic meta-analysis.

**Table 3** contains information on several components used in the diagnostic analysis, including the SEN, SPE, and AUC. The sample sizes ranged from 30 to 192. Applicability issues and risk of bias were considered when evaluating the quality of the included studies, and the findings demonstrated that the included studies were of high quality (**Figure 2**). **Table 4** shows the correlations between circRNAs and clinicopathological features.

### 3.3 Prognosis analysis

**Figure 3:**
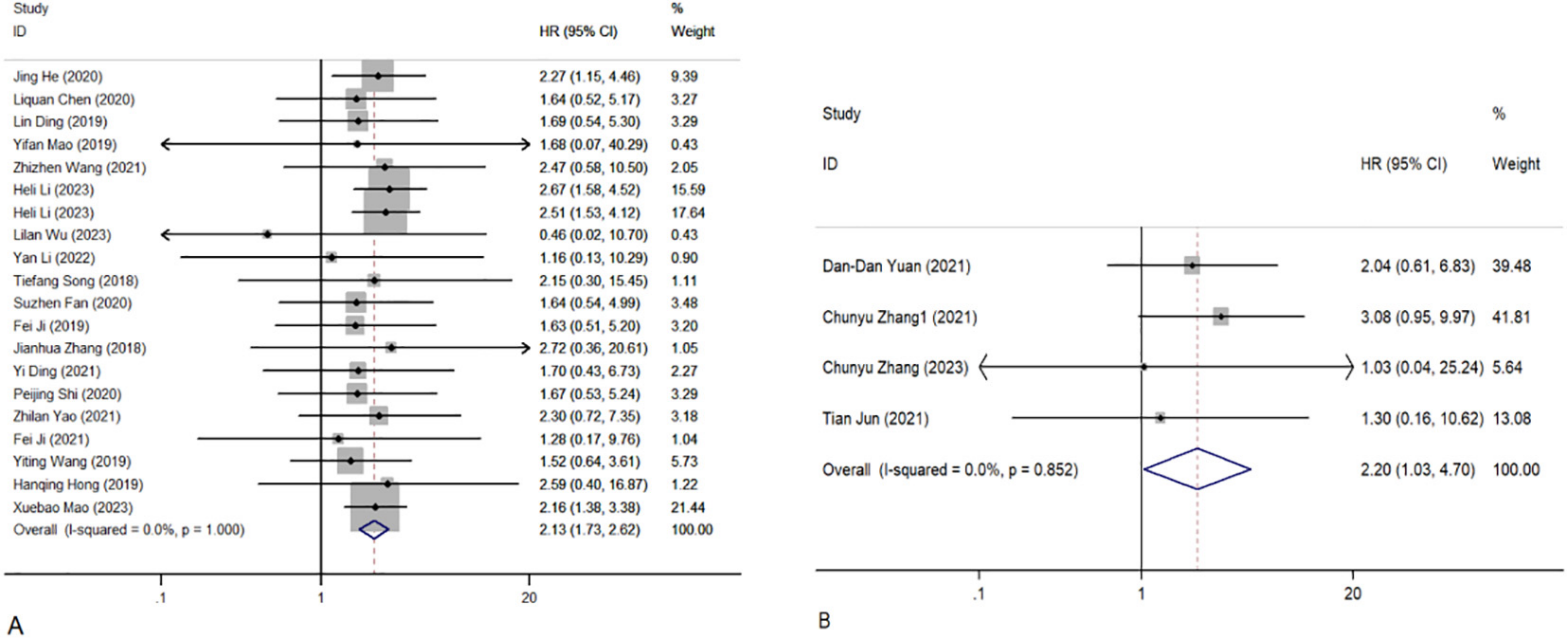
Forest plots of the OS of CC patients for circRNAs. **(A)** Upregulated circRNAs. **(B)** Downregulated circRNAs. HR, hazard ratios.

**Figure 4:**
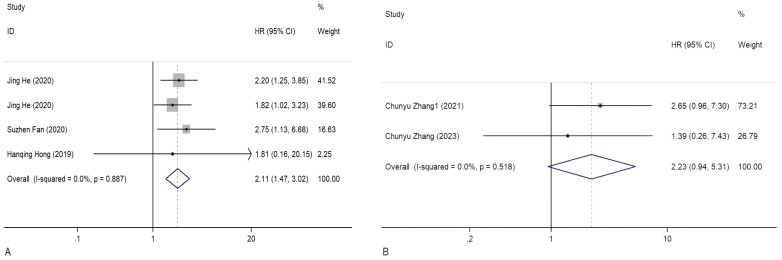
Forest plots of the DFS of CC patients for circRNAs. **(A)** Upregulated circRNAs. **(B)** Downregulated circRNAs. HR, hazard ratios.

Overall, 1,689 people from 23 studies were included in the prognosis meta-analysis. Fixed-effects models (I^2^ = 0.0%, *P* = 1.000) were used to determine the function of elevated circRNAs in CC prognosis. The findings showed that overexpression of carcinogenic circRNAs was associated with a poor prognosis (HR = 2.13, 95% CI: 1.73–2.62, *P* < 0.001) (**Figure 3A**). Concurrently, a significantly poorer prognosis for cancer patients with downregulated tumour-suppressor circRNAs was linked to them (HR = 2.20, 95% CI:1.03–4.70, *P* = 0.042). A fixed-effects model was used because of the lack of heterogeneity among the trials (I^2^ = 0%, *P* = 0.852) (**Figure 3B**). Furthermore, we summarised and analysed the correlation between circRNAs and DFS in patients with CC. We found that overexpression of oncogenic circRNAs affects the DFS of cervical cancer (HR=2.11, 95% CI=1.47–3.02, *P*<0.001), while downregulation of oncogenic circRNAs is not statistically significant for the DFS of cervical cancer (HR=2.23, 95% CI=0.94–5.31, *P*=0.070). We used fixed-effects models for our analysis (I^2^ = 0.0%, *P* = 0.887; I^2^ = 0.0%, *P* = 0.518). The precise numerical values are shown in **Figure 4**.

**Figure 5:**
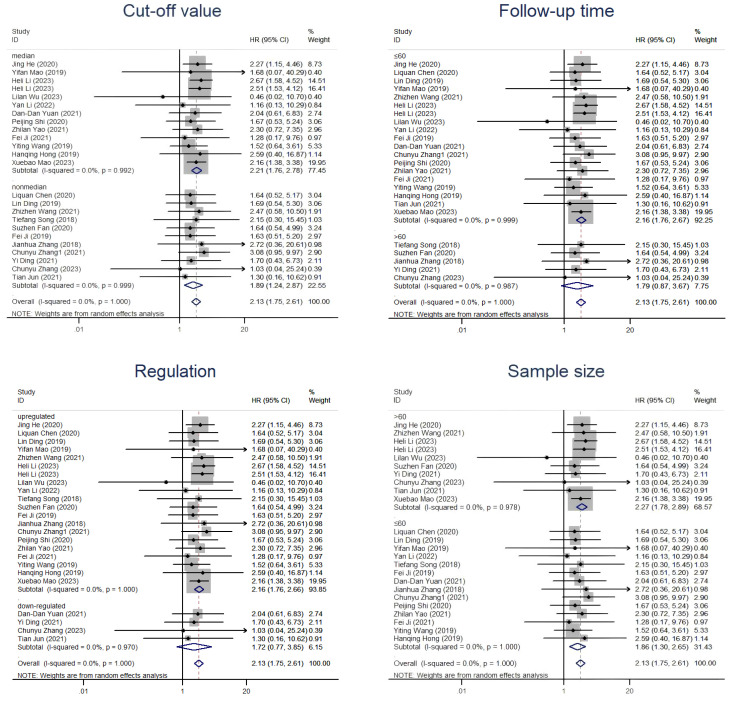
Subgroup analyses of OS for circRNAs, stratified by cut-off value, follow-up time, regulation and sample size.

**Figure 6:**
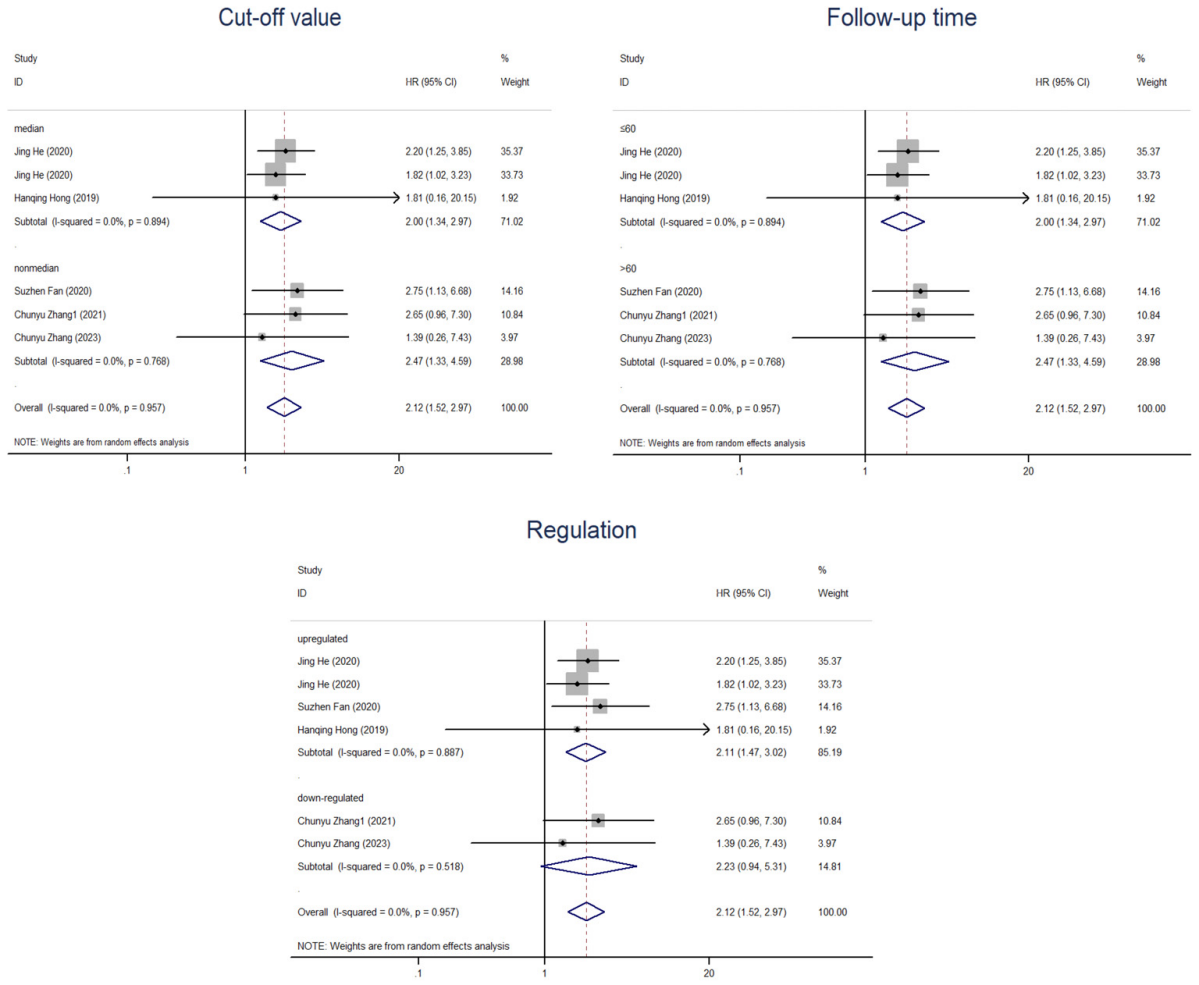
Subgroup analyses of DFS for circRNAs, stratified by cut-off value, follow-up time and regulation.

In the subsequent subgroup analysis, we further found that follow-up time ≤60 months (HR=2.16, 95% CI=1.76-2.67) and upregulation of circRNAs (HR=2.16, 95% CI=1.76-2.66) were risk factors affecting OS (**Figure 5**). However, the two subgroups of cutoff values and sample sizes did not seem to significantly change the relationship between circRNAs and CC prognosis. In the subgroup analysis related to DFS, we found that only the upregulation of circRNAs (HR=2.11, 95% CI=1.47-3.02) was a risk factor for prognosis, while there was no strong association between the follow-up time and the cut-off value with CircRNAs (**Figure 6**). Nevertheless, it should be noted that this conclusion is based on the statistical analysis of existing data, and the actual biological significance may require further experiments and research to verify.

### 3.4 Diagnosis analysis

**Figure 7:**
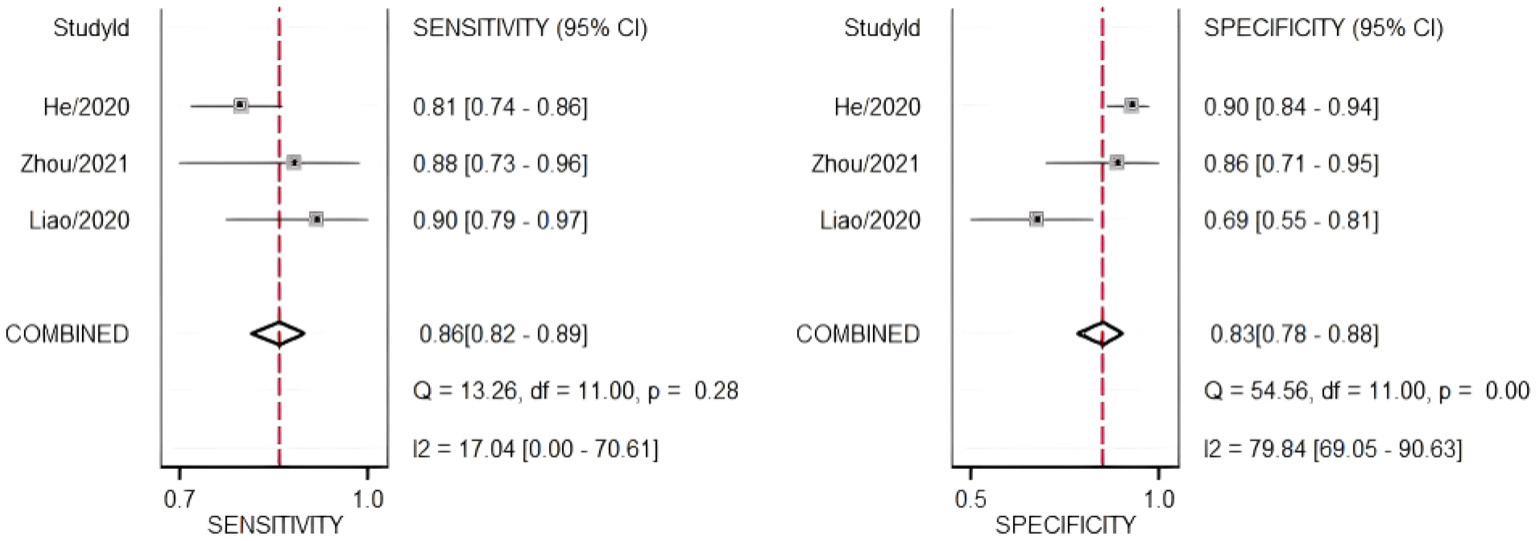
Forest plots of summary sensitivity and specificity to illustrate the diagnostic value of circRNAs for CC.

**Figure 8:**
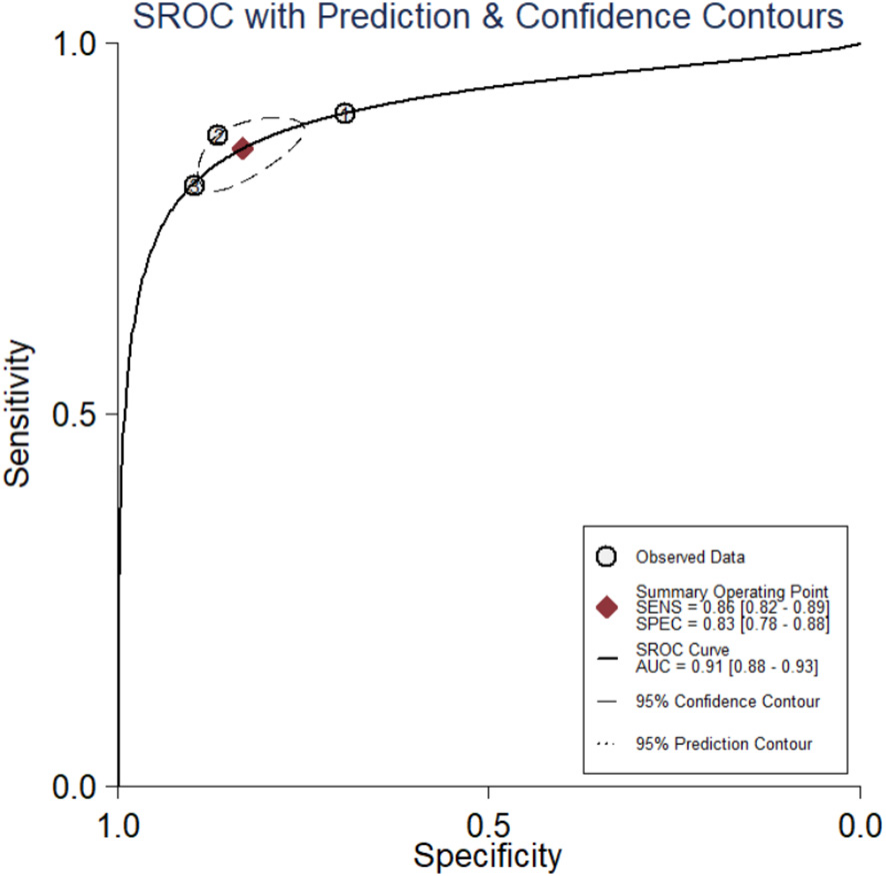
The summary receiver operating characteristic (SROC) curve based on circRNAs for diagnosis analysis. ROC, receiver operator characteristic.

The diagnostic meta-analysis included 274 eligible patients selected from three qualifying trials. The combined SEN and SPC were computed to assess the diagnostic effectiveness of the circRNAs, and the results are presented in **Figure 7**. Analysis of the combined data revealed a sensitivity of 0.86 (95% CI:0.82–0.89) and a specificity of 0.83 (95% CI:0.78–0.88). Furthermore, the study of the SROC curve revealed an AUC of 0.91 (95% CI:0.88–0.93, **Figure 8**). These findings indicated that the tested circRNAs have diagnostic importance in CC, demonstrating high sensitivity and specificity.

### 3.5 Clinicopathological parameters

In addition to their function in assessing the prognosis of patients with CC and aiding in the diagnosis of cancer, circRNAs are closely associated with other clinicopathological markers in patients with cancer. We compiled data from 15 studies, totalling 765 participants, for our meta-analysis to quantify the association between circRNAs and clinicopathological features of CC (**Table 4**). We then summarised the information contained in each article among the 15 studies that included the same influencing factors.

Elevated expression of cancer-causing circRNAs was associated with negative clinical features (tumour size: OR=0.425,95% CI: 0.267–0.676; FIGO stage: OR = 0.315, 95% CI: 0.224–0.443; lymph node metastasis: OR =2.975, 95% CI: 1.816–4.872). Reduced expression of tumour suppressor circRNAs was similarly associated with poorer clinical outcomes (tumour size: OR = 0.310, 95% CI: 0.102–0.941; FIGO stage: OR = 0.231, 95% CI: 0.101–0.527; lymph node metastasis: OR =2.430, 95% CI: 1.156–5.110). In addition, patient age and the degree of tumour differentiation were not statistically significant in relation to circRNA expression.

### 3.6 Publication bias and sensitivity analysis

**Figure 9:**
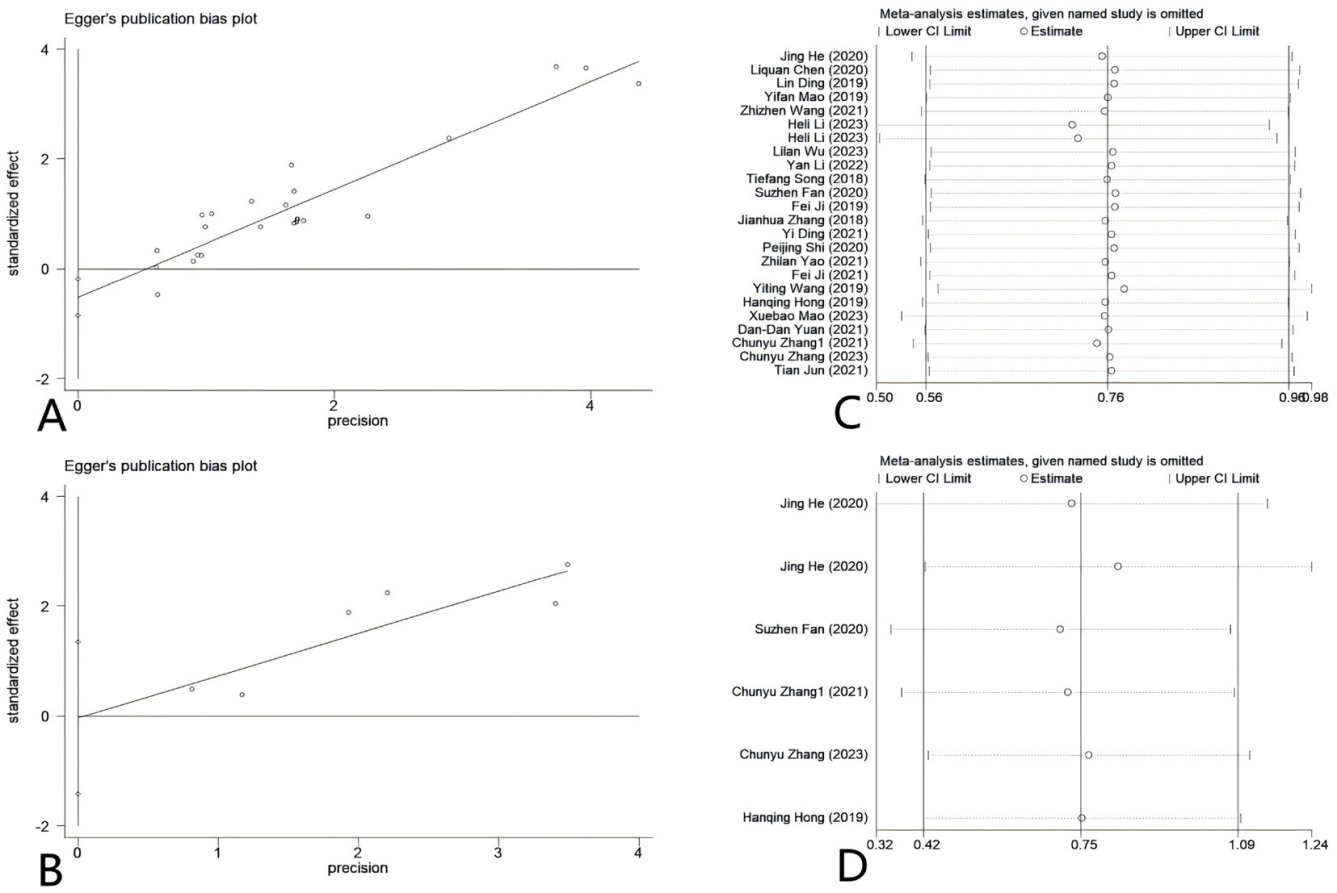
**(A)** Egger’s tests of OS (*P* = 0.003). **(B)** Egger’s tests of DFS(*P* = 0.942). **(C)** Sensitivity analysis of OS. **(D)** Sensitivity analysis of DFS.

Deeks’ funnel plot asymmetry test was used to assess possible publication bias in the diagnostic meta-analysis. The results indicated the absence of publication bias (*P* = 0.80) (**Supplementary Figure S1**). Egger’s tests were used to examine the prognostic markers of CC in relation to circRNA expression and to assess the presence of potential publication bias in the operating system. **Figure 9A** demonstrates that the results of Egger’s tests for OS showed a P value of 0.003, suggesting the presence of publication bias in the included studies. For DFS, the result showed *P* = 0.942 for Egger’s test, indicating no significant publication bias (**Figure 9B**). After implementing the trim-and-fill method, the adjusted effect size was recalculated and the funnel plot was re-evaluated for symmetry. Despite adjusting for potential publication bias, the overall effect size remained significant (*P* < 0.05), suggesting that the initial findings were not significantly influenced by the presence of publication bias (**Supplementary Figure S2**). Concurrently, our study showed that the combined findings of the prognostic meta-analysis remained consistent when subjected to sensitivity analysis (**Figures 9C, D**).

## 4 Discussion

Cervical cancer (CC) is a significant contributor to the global mortality from gynaecological cancers. The primary aetiology of CC is a persistent infection with high-risk HPV types. Cancer development is influenced by a complex interplay of genetic and epigenetic factors that facilitate disease progression. The most common histological subtype of CC is squamous cell carcinoma (SCC), which constitutes approximately 80% of all cases, followed by adenocarcinoma (AC), which accounts for approximately 20%. The implementation of robust CC screening programs and widespread HPV vaccination in developed countries have resulted in a considerable reduction in the incidence and mortality of SCC (43). However, a contrasting pattern has been observed for AC and SCC, the incidence of which has increased over the past three decades. This increase may be due to the limitations of traditional cytological screening methods, which are unable to effectively detect these subtypes, given their location deep within the cervical canal (44, 45).

The current diagnostic and treatment landscape for CC is multifaceted, and encompasses surgical interventions, radiotherapy, chemotherapy, emerging immunotherapies, and targeted therapies. Despite these advances, challenges such as the side effects of chemoradiation, limitations of immunotherapy, and the need for more effective treatments, particularly for metastatic disease, persist. The standard of care often involves concurrent chemoradiation of locally advanced tumours and a combination of chemotherapy with bevacizumab for metastatic disease. However, these treatments can be limited by toxicity and immunosuppression (46).

In the quest for novel therapeutic approaches, circRNAs have emerged as promising candidates for CC research. CircRNAs are a class of non-coding RNAs that exhibit distinctive expression patterns in eukaryotic transcriptomes owing to their covalently closed continuous-loop topology. They have been found to play a role in various cellular processes including cell growth and development, and their abnormal expression has been associated with CC development (14). The potential use of circRNAs in CC lies in their ability to serve as diagnostic biomarkers and therapeutic targets. They regulate cell proliferation, migration, invasion, and apoptosis. Their unique profiles in CC offer opportunities for early detection and personalised treatment strategies (47). As our understanding of the role of circRNAs in CC increases, these molecules may provide new avenues for improving patient outcomes and developing effective treatments.

Recently, several studies have focused on the function of circRNAs. However, there is a notable absence of relevant meta-analyses examining circRNA expression in CC. In previous meta-analyses, only one study has detected an association between circRNAs and CC. Liu et al. (48) identified circRNAs as potential biomarkers for both cervical and ovarian cancers, demonstrating their diagnostic and prognostic value. However, the scope of their study, although comprehensive, resulted in a less focused analysis of CC and did not incorporate clinicopathological data. Recent reviews have explored the role of circRNAs in CC development and progression. Chaichian et al. (49) highlighted the substantial influence of circRNAs on CC progression, and Najafi et al. (50) expanded the discourse to include the prognostic, diagnostic, and therapeutic implications of circRNAs in clinical settings. However, the absence of quantitative data indicates that these findings require empirical validation.

A total of 1983 patients with cancer from 27 suitable studies were included in this analysis in accordance with the specified inclusion and exclusion criteria. These studies included three studies that focused on diagnosis, 23 focused on prognosis, and 15 focused on clinicopathological aspects. Regarding prognostic risk factors, 19 circRNAs were linked to OS in patients with CC, whereas three circRNAs were linked to DFS. Overall, increased expression of oncogenic circRNAs is linked to a much higher risk of death in patients, being 2.13 times greater than those without such increase (95% CI: 1.73–2.62). Similarly, the downregulation of tumour-suppressor circRNAs confers a 2.20-fold increased risk of death, albeit with a wider confidence interval reflecting less certainty (95% CI: 1.03–4.70). In our prognostic meta-analysis that focused on OS, Egger’s test indicated the potential for publication bias, which merits further examination. This bias indicates that studies with insignificant or less impressive results may be less likely to be published, which could potentially bias the pooled results. Nevertheless, the application of the trim-and-fill method to account for this asymmetry did not yield a statistically significant difference in the results, indicating that despite the potential influence of publication bias, it had a minimal effect on the overall findings of our study.

In the context of CC diagnosis, a limited number of three studies were identified, which reported an AUC of 0.91 for circRNAs as potential diagnostic biomarkers. These studies indicated a sensitivity of 0.85 and a specificity of 0.83, suggesting that circRNAs can be used to effectively distinguish between healthy individuals and those with CC. In addition, some studies have highlighted the significant role of circRNAs in screening for cervical intraepithelial neoplasia (CIN). Luo et al. (51)analysed the temporal transcriptomic landscapes of mRNAs and circRNAs, identified functional circRNAs in cervical squamous cell carcinoma (CSCC), and improved our understanding of the pathogenesis and molecular biomarkers of CSCC and high-grade squamous intraepithelial lesions (HSIL). Zhou et al. (52) found that ciRS-7 could be used to discriminate between CC patients and healthy controls, as well as between CC and CIN patients, demonstrating great potential in clinical diagnosis. In conclusion, circRNAs may serve as potential biomarkers of CC and CIN. Therefore, circRNAs can be considered candidate biomarkers for screening CIN, including atypical hyperplasia. However, further studies are needed to verify the accuracy, sensitivity, and specificity of these circRNAs as clinical diagnostic tools.

In conclusion, we conducted a meta-analysis of the clinicopathological data from 765 patients with CC who participated in 15 different investigations. Notably, tumour size, FIGO stage, and lymph node metastasis were significantly associated with the expression of both upregulated and downregulated circRNAs, suggesting their integral involvement in tumour progression and metastatic potential.

Changes in circRNA expression are noted in response to chemotherapy, radiation therapy, and immunotherapy. For example, by reducing the expression of HSPB1, crVDAC3 induces ferroptosis in breast cancer cells, thereby mediating the resistance of HER2-low breast cancer to trastuzumab-deruxtecan (53). A growing body of evidence suggests that circRNAs may serve as prognostic biomarkers for predicting clinical response to cancer chemotherapy. However, more detailed experimental research is required to support their translational applications in therapy and prognosis (54). In addition, studies have shown that changes in the tumour microenvironment such as hypoxia, immune cell infiltration, and the presence of secreted factors can potentially affect the expression and function of circRNAs, thereby affecting the accuracy of circRNA detection (55). Therefore, it is necessary to consider the complexity and dynamic changes in the tumour microenvironment when performing circRNA detection to ensure the accuracy and reliability of the results.

These characteristics of circRNAs will not only be able to guide clinical decisions but also act as therapeutic targets or agents, paving the way for new treatment interventions. Due to the stable and non-degradable nature of circRNAs, several circRNA vaccines have been synthesised and tested both *in vitro* and *in vivo (* 56). As research progresses, circRNAs are expected to open new chapters in precision medicine and become important platforms for future clinical diagnoses and treatments.

The results of this meta-analysis should be interpreted with caution owing to several constraints. First, all the analysed research originated in China. Hence, the generalisability of our findings to other populations may have been affected. Second, the diagnostic analysis was affected by the limited dataset of only three studies, and more data are needed to validate the results. Furthermore, the lack of explicit hazard ratio (HR) data in some studies required data extraction from the Kaplan-Meier (KM) curves, which could have introduced bias. Finally, the study did not incorporate bioinformatics prediction or validation of potential pathways, which represents an area for future enhancement. In the subsequent studies, we aimed to bridge this gap by integrating computational analyses to delineate the underlying mechanisms of circRNAs in CC. This approach refined our understanding of their roles and potentially uncovered novel targets for intervention, thereby enriching the existing body of research.

The results of our study indicate a significant correlation between circRNA dysregulation in patients with CC and key clinical parameters, including diagnosis, prognosis, and tumour characteristics. These findings have profound implications for biological understanding and clinical management of CC. Future research should elucidate the mechanistic roles of circRNAs as this may aid in the identification of novel therapeutic targets and foster innovative diagnostic and treatment strategies for CC.

## Data Availability

The original contributions presented in the study are included in the article/**Supplementary Material**. Further inquiries can be directed to the corresponding author.
