# Supplementary figures and images for: Prognostic and diagnostic value of circRNA expression in cervical cancer: a meta analysis
Source: Front Oncol. 2025 Jan 13;14:1488040. doi: 10.3389/fonc.2024.1488040 (PMC11769824; doi:10.3389/fonc.2024.1488040)

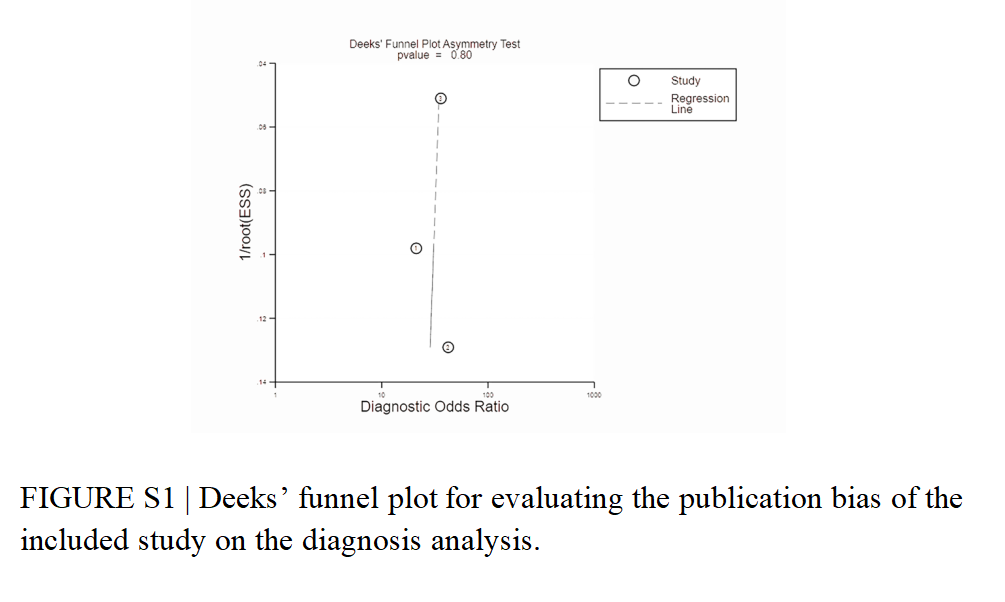

Supplement: Supplementary file 1 [file Image1.tif]

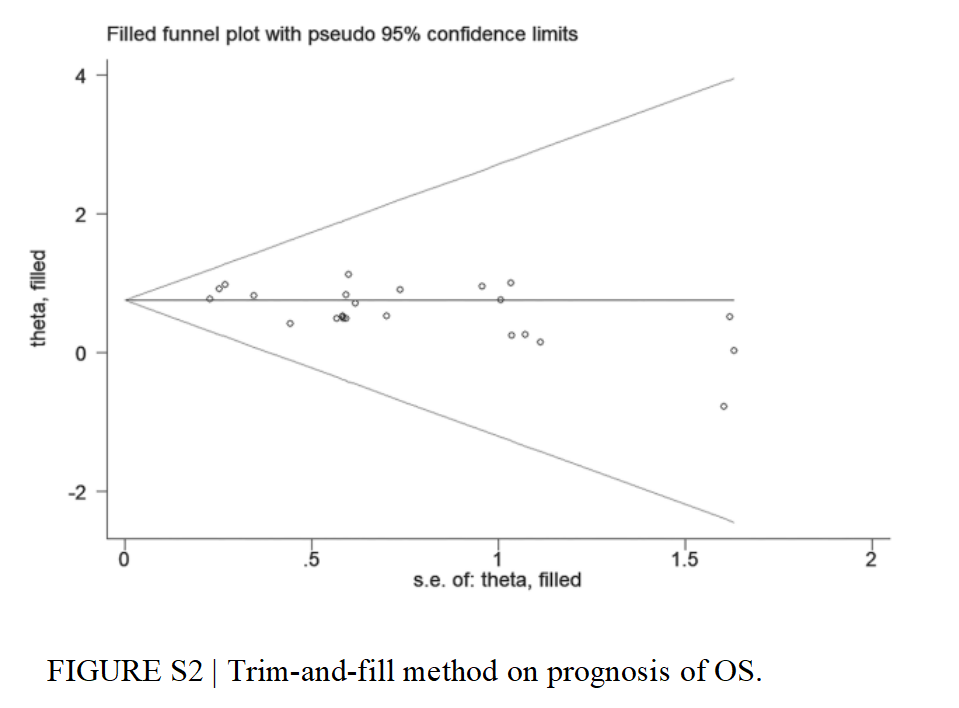

Supplement: Supplementary file 2 [file Image2.tif]
